# A Systematic Review of the Effect of Arts-Based Interventions on Patient Care in Nigeria

**DOI:** 10.7759/cureus.32883

**Published:** 2022-12-23

**Authors:** Emmanuel O Oladeji, Constantine Ezeme, Seun Bamigbola

**Affiliations:** 1 Department of Surgery, University College Hospital, Ibadan, NGA; 2 Department of Surgery, Alimosho General Hospital, Lagos, NGA

**Keywords:** nigeria, alternative therapy, dance, visual arts, music, art therapy, art in health, art in medicine

## Abstract

The utilization of art for therapeutic purposes in the formal healthcare setting is gradually gaining prominence in Nigeria. However, there is a paucity of evidence on the effectiveness of these interventions. Therefore, we explored the pooled effect of the various arts-based interventions in managing clinical disorders in hospitalized and out-patients in Nigeria. An electronic search of PubMed, African Journal Online, Web of Science, Google Scholar, Cochrane Library, and Scopus databases was carried out from the inception of the databases to October 31, 2021. Three researchers using Rayyan QCRI software independently screened and de-duplicated the identified studies. Eight eligible studies were selected for this review, with a total of 541 participants. The earliest study was published in 2012. Seven of the eight studies were conducted in the Southern part of Nigeria. There were four quasi-experimental studies, two randomized controlled trials, and two comparative cross-sectional studies. The predominant art forms were music (three) and dance/movement (three), followed by visual art (two). The groups of patients in the identified studies were managed for mental health problems (two), hypertension (two), Parkinson’s disease (one), spinal cord injury (one), autistic spectrum disorder (one), and chronic back pain (one). In all the studies, the art-based intervention significantly improved the overall outcome of the patients. The findings of the available studies have proven to yield a significant positive outcome in managing different health conditions. However, there is a need to conduct more high-quality research in this field in Nigeria.

## Introduction and background

The use of art to enhance healthcare has grown remarkably in the last few decades. It is an active field of practice that unifies a professionally diverse community driven by utilizing art's immense potential to foster improved patient experience in various forms [1]. Art in health comprises many subfields and affiliated fields and is known by a variety of descriptors. Regardless, the common theme is integrating creative experiences like music, dance, theatre, literature, visual art, architecture, and interior design to improve patient clinical, experiential, and holistic outcomes [1-4]. Without making a claim that art alone can heal an illness or replace pharmacotherapy or input of medical or mental health professionals, arts-based interventions present a less expensive, accessible, convenient option to healthcare practice, devoid of known side effects and promote health in a wholistic sense as defined by WHO [5,6].

The therapeutic use of arts-based interventions in managing patients in formal healthcare settings in Nigeria is gradually gaining prominence [7]. Specific applications in hospitals, hospices, and homes for the elderly, include bedside activities facilitated by professional artists, art installations and performances, and community-based art programs designed to improve wellness and health [7]. However, despite the recognition of emerging benefits in children suffering from sickle cell disease or cancer, patients with mental health problems, people suffering from chronic pain and chronic medical conditions, and the growing geriatric population [7-15], there has been no systematic review focused on evaluating the evidence of arts for health activities in Nigeria. This systematic review aims to appraise and synthesize existing empirical studies that have evaluated the impact of arts on health activities in clinically diagnosed Nigerians who were managed for specific health conditions using arts-based interventions. The outcome measures evaluated were improvement in health, well-being, and quality of life post-intervention. Evidence generated from this review will serve to guide practice of art in health practitioners in Nigeria and the African ecosystem at large and instruct further research directions.

## Review

Methods

Study Registration

Prior to conducting the review, a protocol was developed and registered with the International Prospective Register of Systematic Reviews (PROSPERO) [16]. The study was conducted in line with the Preferred Reporting Items for Systematic Reviews and Meta-Analyses Protocol (PRISMA-P) statement using the PICOS framework [17,18].

Databases and Search Strategy

The electronic search of PubMed, African Journal Online, Web of Science, Google Scholar, Cochrane Library, and Scopus was conducted using a developed search strategy from the databases' inception to October 31, 2021. Original studies retrievable in English were included in the review. Additional hand-search of reference lists from retrieved articles was also conducted.

Eligibility Criteria

Articles were eligible for inclusion in the review if they were experimental or quasi-experimental, conducted among hospitalized or out-patients with a confirmed medical diagnosis who received art-based intervention as an adjunct to healthcare and retrievable in the English Language. Studies were excluded if they failed to provide details on the source of the study groups, were conducted among healthy persons with no medical diagnosis, were not retrievable in the English language, or fell into one of the categories of reviews, descriptive studies, comments, or letter to the editor.

Interventions and Comparator

Arts-based interventions involve the use of music, painting, dance/movement, expressive arts, poetry/expressive writing, drama, psychodrama, or a combination of these as an adjunct to the care of hospitalized or out-patients. The primary outcome measures were changes in the health condition of participants.

Data Screening

The title and/or abstract of all studies returned from the different databases were independently and blindly screened by the first two authors using the Rayyan QCRI software package. The screening identified studies that met the inclusion criteria. Discrepancies during the screening process were resolved by the third author, who had not been involved in the earlier screening process.

Data Extraction and Management

All relevant data were extracted from the included studies using Microsoft Excel and based on the PICOS framework by two reviewers [18]. The data extracted included the first author's name, year of publication, nationality, the language of publication, study design, sample size, patients' social demographic and clinical characteristics, type of art form, and outcome measures, and the statistical analysis was done. The suitability of the available data for meta-analysis was accessed after statistical analysis [19,20].

Results

**Figure 1:**
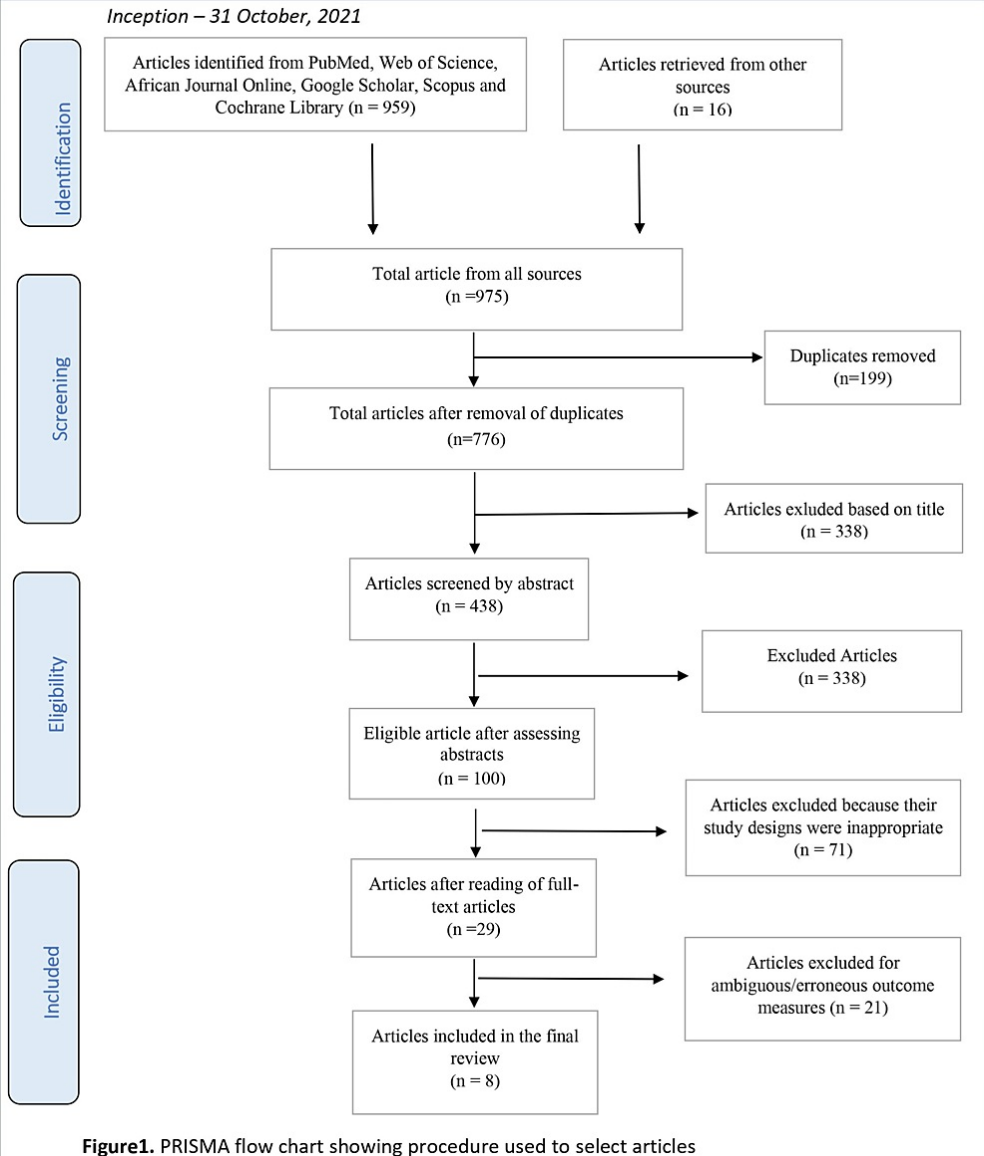
PRISMA flow chart showing procedure used to select articles. PRISMA: Preferred Reporting Items for Systematic Reviews and Meta-Analyses.

The summary of the article selection process is shown in Figure 1.

The initial search produced a total of 975 papers. Screening of titles, removal of duplicates, and non-English language papers led to the exclusion of 875 articles. A review of the remaining 100 article abstracts resulted in the exclusion of an additional 71 articles because of inappropriate study design. Of the 29 remaining articles, 21 were further excluded due to erroneous/unclear outcome measures. Eight articles were included in the final analysis. The characteristics of the eight included studies are summarized in Table 1.

**Table 1:** Summary of characteristics of the included studies.

S/N	Authors	Year	Region (state)	Sample Size (N= 541)	Study Design	Setting	Art form used	Duration of intervention (weeks)	Medical Condition	Outcome
1	Osimade OM [8]	2020	Oyo	65	Quasi-experimental: Pre & post	Community setting	Music	Eight	Depression	Improvement in symptoms of depression
2	Okafor UA et al. [11]	2012	Lagos	30	Randomised controlled trial (RCT)	Out-patient setting in a tertiary hospital	Dance/movement	Six	Chronic low back pain	Less pain intensity, improved functional disability and quality of life
3	Abodunrin JA [12]	2019	Oyo	60	Comparative cross-sectional	Unclear	Visual arts: painting	Not stated	Parkinson’s Disease	Improved sense of individual value, better self-expression/level of confidence, enhanced muscular relaxation, improved eye-hand coordination and memory
4	Aweto HA et al. [13]	2012	Lagos	38	RCT	Out-patient setting in a tertiary hospital	Dance/movement	Four	Systemic hypertension	Reduction in resting systolic blood pressure and resting diastolic blood pressure (additionally, reduction in resting heart rate, maximum heart rate and estimated oxygen consumption)
5	Akinwumi RO and Mojoyinola JK [14]	2015	Lagos	120	Quasi-experimental: Pre & post	Rehabilitaion center	Music	Eight	Spinal cord injury	Reduction in pain; improvement in mood and spiritual well-being, and overall quality of life.
6	Ezenwa M [15]	2012	Ebonyi	10	Quasi-experimental: Pre & post	Out-patient setting in a tertiary hospital	Music	(One session)	Systemic hypertension	Reduction in systolic and diastolic blood pressure
7	Quadri OO [21]	2015	Lagos	20	Comparative Cross-sectional	Care home for children with special needs	Visual arts: paper weave, okra printing, papier mache, painting, drawing	Four	Autism	Improved self-expression, communication and socialisation
8	Salihu D et al. [22]	2021	Borno	198	Quasi-experimental: Pre & Post	Internally displaced persons (IDP) camp	Dance	Eight	Depression	Improvement in depressive symptoms, (as well as stress, and anxiety)

Characteristics of Included Studies

**Figure 2:**
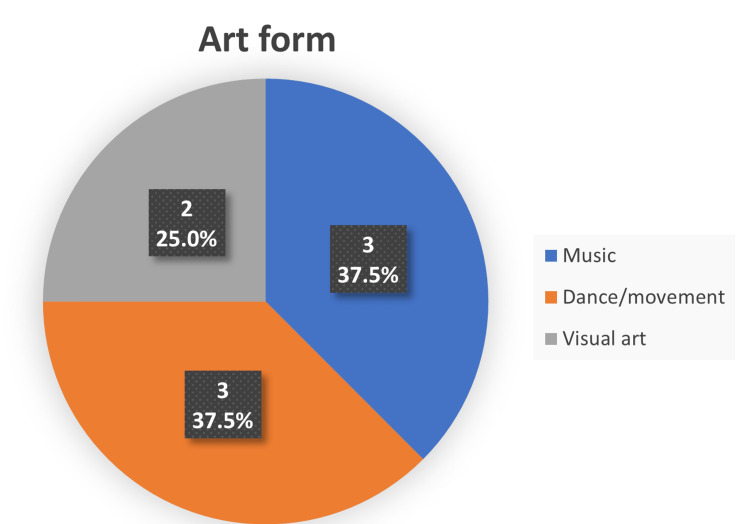
Proportion of studies by art form utilised (%).

This systematic review encompasses data from 541 participants included in eight studies. The characteristics of the included studies are summarized in Table 1. The publication dates of the included studies ranged from 2012 to 2021, with most of the studies (62.5%) published on or before 2015. The included studies varied in terms of their study design. A total of 50% of the papers used quasi-experimental, 25% randomized controlled trials (RCT), and 25% comparative cross-sectional study designs. All the included studies were intervention studies and conducted prospectively. Music and dance were the most commonly adopted art forms, each accounting for 37.5% of the studies. In contrast, visual art forms, including painting, drawing, sculpture, and crafts, were utilized in the remaining 25% (Figure 2).

Location and Setting

All the included studies were conducted in Nigeria. Three-fourths were from two states in the South-Western region of the country, dominated by Lagos (50%) [11,13,14,21], while the remaining 25% were from Oyo state [8,12]. A total of 12.5% of the studies were carried out in Ebonyi state and Bornu state, from the South-Eastern and North-Eastern regions of the country, respectively [15,22]. In half of the art-based activities, participants were recruited from a healthcare facility, three (37.5%) from an out-patient setting of a tertiary hospital [11,13,15], and one (12.5%) from the rehabilitation center for spinal cord injured patients [14]. A total of 25% of the studies were carried out in community-based settings, the first at an internally displaced persons (IDP) camp [22] and the second in a rural community among elderly individuals with a diagnosis of depression [8]. One other study took place in a care home for children with special needs [21]. The last study was unclear about the setting of the art intervention, and the available information was insufficient to deduce the environment [12].

Characteristics of Arts-based Interventions

**Figure 3:**
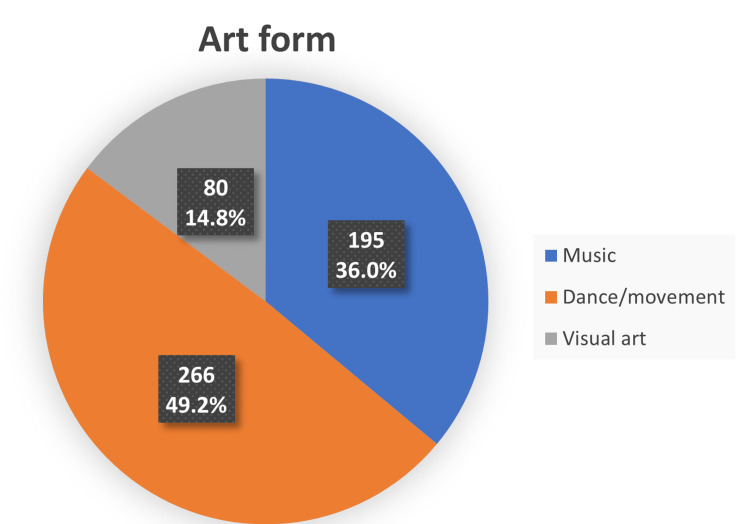
Art form used by participants (%).

The type of arts-based interventions utilized in the included studies varied in structure and content between musical, dance-related, and visual arts activities. As presented in Figure 3, 195 participants from three studies were exposed to music in various forms [8,14,15]. 

A vast majority were spinal cord-injured patients who underwent music therapy at a rehabilitation center [14]. Similarly, Ezenwa M investigated the use of music in the management of systemic hypertensive patients [15], while Osimade OM explored the effect of music intervention in geriatric participants with depression [8]. Classical music was the genre adopted by Ezenwa M for all participants [15], in contrast to Osimade OM, who tailored the musical choices to participants' preferences [8]. This was accomplished by requesting participants to provide five favorite musical performances while inviting family members into the sessions to create a relatable ambiance. The dimensions of music therapy offered by Akinwumi RO and Mojoyinola JK were not described in sufficient detail [14]. Three interventions focused on dance, involving a total of 266 participants [11,13,22]. In a quasi-experimental study, Salihu D et al. accessed the impact of African Circle Dance (ACD) on participants who had depression [22]. Sessions were facilitated by a dance specialist who employed a band of African musicians and instrumentalists to play African music. At the same time, the participants partnered and performed the traditional Maliki dance in a circle. Both Okafor UA et al. and Aweto HA et al. adopted dance as an intervention using a randomized control study design [11,13]. While the former used a dance protocol that involved aerobic dance [11], rolling exercises, and specific back extension exercises in lying and standing postures in patients with chronic back pain, the latter assessed the therapeutic value of dance movement therapy (DMT) in hypertensive individuals [13]. The authors defined DMT as ''the psychotherapeutic use of movement and dance for emotional, cognitive, social, behavioral and physical conditions''. The study group was exposed to DMT as an adjunct to pharmacotherapy with anti-hypertensives. The two other interventions implemented visual art forms [12,21]. In the first, children with autism were encouraged to engage in visual art activities with varying degrees of complexity. This included paper weaving with luminous sugar paper, okra printing using poster color, creating functional shapes and images from paper, making paper sculptures, and painting and drawing activities [21]. In the second intervention, the therapeutic benefit of painting engagement on emotional, somatic, and affective symptoms was evaluated in patients with a diagnosis of Parkinson's disease [12].

Characteristics of Included Population

A total of 541 participants were included in this review, 20 children inclusive. The sample size varies widely from 10 to 128, with a median of 49. A total of 75% of the studies reported mean age or age range of participants, with an overall range of 18-75 years, while others reported population using descriptors such as 'boys,' 'girls,' or 'adults' [14,21]. Only one of the studies was conducted on children, which included ten boys and ten girls; however, there was no clear information about their exact age distribution [21]. The gender of those who partook in the studies was reported in 75% of the papers, with females representing 53.9% of the cumulative sample and male participants making up 46.1%. One study reported more males than females [12], two reported equal numbers [15,21], and three reported more female than male subjects [11,14,22]. A total of 50% of the studies explored art-based interventions in individuals with a diagnosis of systemic hypertension or depression. Ezenwa M investigated ten patients with systemic hypertension using music intervention [15], while Aweto HA et al. implemented dance intervention in 38 patients with similar diagnoses [13]. Osimade OM featured an elderly cohort of 65 participants with depression [8], in contrast to Salihu D et al., whose intervention was directed towards a younger group of 198 IDPs, with 91.9% in the 18-49 age group [22]. Participants in the remaining studies underwent art activities for low back pain [11], Parkinson's disease [12], autistic spectrum disorder [21], or spinal cord injury [14], with a population size of 30, 60, 20, and 120 participants, respectively.

Duration and Intensity of Intervention

There is a wide variation in the overall duration of interventions delivered across the different studies, ranging from one session to 16 sessions spanning a period of eight weeks. For example, four weeks duration was represented in three studies [13,22], six weeks in one study [11], and eight weeks in three studies [8,14,22]. In terms of the duration of time spent per session, the least reported was thirty minutes, and the longest was two hours. The frequency and intensity varied from weekly sessions to three times a week. However, the details of the duration and intensity were not reported in sufficient detail in some studies.

Outcomes and Outcome Measures

The reported outcomes and outcome measures varied significantly based on the clinical diagnosis of interest, the art form utilized, and the objectives of the respective study. Two studies assessed the impact of art activities on hypertension, with the objective of evaluating the art interventions' effectiveness in reducing systolic and diastolic blood pressure [13,15]. Additionally, Aweto HA et al. investigated the effect of the intervention on resting heart rate, maximum heart rate, and estimated oxygen consumption [13]. The pre and post-exposure readings of the systolic and diastolic blood pressure of the participants were manually taken by research assistants using stethoscope and mercury sphygmomanometer. The resting and maximum heart rates were similarly measured using a stethoscope, and the estimated oxygen concentration was calculated by the Uth-Sørensen-Overgaard-Pedersen estimation. While Ezenwa M took a quasi-experimental approach with a relatively small sample size [15], Aweto HA et al. used RCT with a larger sample, and both confirmed the effectiveness of the art intervention in lowering blood pressure [13]. Two studies validated the benefit of art engagements in managing depression using a quasi-experimental design [8,22]. The screening instruments used for depression were the geriatric depression scale or depression anxiety stress scale (DASS). The focus of the first was to investigate the effectiveness of music intervention on geriatric depression [8]. The author adopted four instruments translated into the local language and used them to assess depressive symptoms pre and post-intervention. The instruments included the Hamilton Rating Scale for Depression (HAM-D), the Big Five Personality Inventory, the Multidimensional Health Locus of Control Scale, and the Geriatric Depression Scale [8]. The second was directed to displaced individuals living in IDP camps who were not only studied for depressive symptoms but also investigated for anxiety and stress as secondary outcomes, using a Hausa language version of DASS [22]. The depression, anxiety, and stress subscale scoring were assessed pre-and post-exposure to intervention. In separate studies, Abodunrin JA and Quadri OO substantiated the value of visual arts in managing Parkinson's disease (PD) and autism, respectively [12,21]. While painting alone was used in the former [12], the latter utilized other forms, which included paper weaving, paper sculpture, papercraft, and okra printing [21]. Abodunrin JA adopted a comparative cross-sectional design to assess the effectiveness of painting in improving fine motor control, hand-eye coordination, self-expression, memory, and overall quality of life, in patients with PD [12]. To investigate which art techniques improved self-expression and communication skills among autistic children, Quadri OO adopted visual art practical performance test as the study instrument. The least complex techniques with clear step-by-step directions were found to be the most engaging and equally effective compared to the more abstract ones [21]. In a group of spinal cord injured patients, Akinwumi RO and Mojoyinola JK demonstrated the merits of music intervention in improving pain control, physical, social, emotional, mental, and spiritual well-being following a quasi-experimental approach, using music therapy assessment and well-being assessment questionnaires [14]. Using dance engagement, Okafor UA et al. similarly prove the value of art intervention in improving pain control, in addition to functional disability and quality of life [11].

Quality of the Reporting Evidence and Evaluation Methods

**Figure 4:**
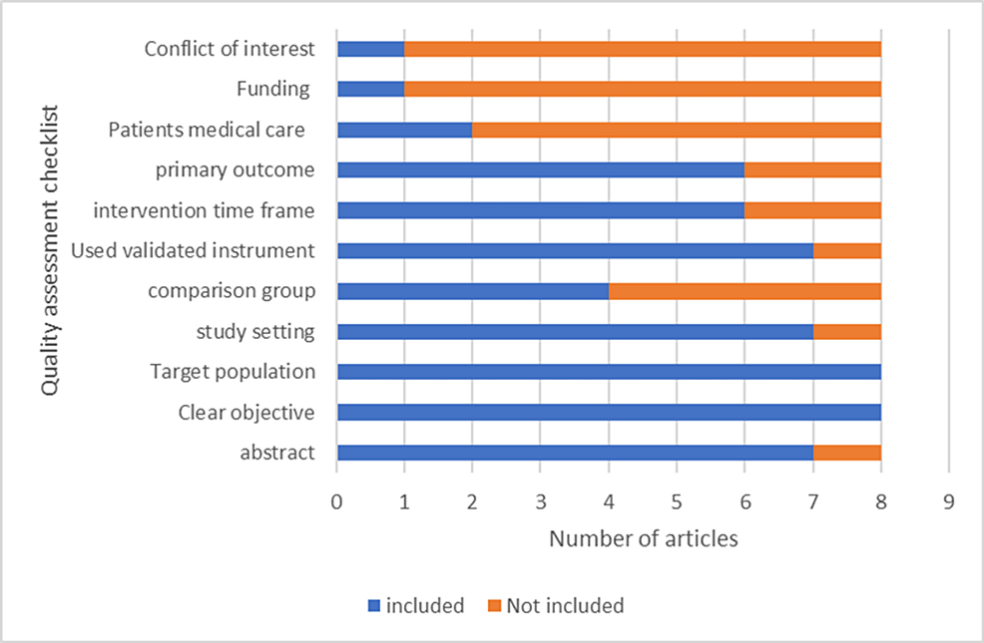
Quality of the reporting evidence in selected studies.

Using a single validated tool to assess the quality of the articles included in this review proved challenging. This is due to the heterogeneity of the articles, study method, study population, intervention, outcome, and outcome measures. However, a checklist was adopted for this purpose (Figure 4). The results of the quality assessment of the studies are summarized in Figure 4.

Overall, only one (12.5%) of reviewed articles met all the checklist criteria [22]. One article met less than 50% of the criteria [12], while six were positive for 50% or more of the checklist criteria [8, 11, 13-15, 21]. Items 11 and 12 (Funding and Conflict of Interest) were reported only by a study [22]. Most of the studies specified the study setting and used validated instruments for outcome measures; one study, however [12], did not report the method employed in the study. Therefore, it was not possible to get the study setting, comparison group, duration of intervention, or whether participants were on any medical treatment from this article. There was no comparison group in three studies [14, 15, 21], and only two of the studies reported the medical treatment the participants were receiving at the time of intervention [13, 21]. Other standards which were not reported in all the articles were the measures taken to eliminate bias in accessing the primary outcome and the limitations in the studies. The limitation imposed by the clinical and methodological diversity of the included studies did not allow for any form of meta-analysis; hence synthesis without meta-analysis (SWiM) was performed [19,20].

Discussion 

The fact that most of the works on art-based therapy in medicine in Nigeria were within the last decade shows that it is a recent addition to the clinicians' therapeutic tools, or at the very least, it recently attained the status of scientific consideration in the healthcare system in Nigeria. This trend does not seem limited to Nigeria; however, Curtis A et al. [23] discovered a similar spike of interest in art within the last decade. The pattern is somewhat different, however. While most of the work on art interventions in Nigeria was done in the first half of the last decade, Curtis A et al. discovered that the interest in art is still on the rise. There is no simple explanation for the declining publications in the second half of the last decade. While the extent to which art is deployed outside experimental settings could not be ascertained, the result of previous studies was positive, and acceptance appeared good to sustain the initial spike of interest. Funding and perennial instability in academics scheduled across the country are other factors that could have affected the research output [24-26]. The inclusion criteria and data processing for this work also inadvertently weeded out recent publications. A genuine decline of interest will not only be worrisome but somewhat erratic, and a huge setback to the progress made so far. 

It is interesting to note that seven out of eight studies considered in this review were conducted in the southern part of the country, with half of the studies within Lagos and another 25% just miles away within the southern part of the country. This may be connected to the heavy presence of art in Lagos. Although art comes in various forms and every region in the country has centuries-old art heritage, industrialization, rural-urban migration, and impressive remuneration for artwork has, however, transformed Lagos into the center of art consciousness in Nigeria [27-30]. It is also good that Lagos is transforming itself from the perceived conduit of art out of Nigeria to establishing itself as a home of art, where art is studied and slowly being integrated into its healthcare system. Even though the Northern part of the country is historically rich in artwork [31,32], only one study came from the Northern part. One would have been tempted to attribute the relative apathy of the Northern scholars to cultural and religious setting of their environment, given that about 85% of the whole participant were exposed to either music or dance, both of which are relatively unpopular forms of art in the North [33-36]. However, this will be fallacious when one realizes that the Southeast, with a similar social and cultural setting to the Southwest, has the same representation in terms of proportion while South-South has none. Also, the attitude of the participants in the study by Salihu D et al. [22] was not shown to be different from the participants in the other seven studies. The simple answer is that eight publications are actually too few to make any scientific sense. Giving weight to the such pattern at this stage is rather precocious. 

Because of the ease of use, music and dance are the most deployed forms of art in the clinical setting. Other art forms are also deployed, and the results are just as positive. More studies showing art-based interventions of different forms of art in a different population, clinical settings, and on a much larger population are, however, needed to make any serious scientific assertions. While the protocol for this review took care of most of the low-quality publications on art-based intervention in clinical practice, it is worthy of note that the quality of the articles differs significantly. Therefore, generalization may be rather naive. Aweto HA et al. and Ezenwa M broke away from the usual application of art in treating neurocognitive disorders by evaluating the impact of art on one of the most popular chronic medical diseases in medicine, hypertension [13,15]. Aweto HA et al. did not only measure the response of the patient blood pressure to art interventions, but they also went a little further in standardizing the response by eliminating some other variables that affect blood pressure in awake patients. The fact that art is cheap, pleasant, and comes in different forms, makes it uniquely interesting as an adjunct or alternative to present-day therapeutic interventions [1,6]. It will be interesting to see how much impact this can make in treating medical diseases on a public health scale. It will be equally fascinating to see future research direction on whether the art has a metered effect on blood pressure and what stage of hypertension it should be recommended precisely. It is also more helpful to make the response quantitative rather than qualitative. This will allow for a predictive outcome while prescribing art as an alternative or adjunct to pharmacologic agents.

Another important variable that is generally of importance to clinicians but omitted by most studies is the onset of action. Physicians generally like to know what the onset of action of any form of therapeutic intervention deployed is. How long it took before the participants started feeling some responses? Were these responses sustained or disappeared after a while? What is the pattern of this response? Did it increase over time, plateau, or decline? What is the intensity of these responses? Does the genre of music affect the response? Is there any difference in the response of artists and passive art enthusiasts? Is there any agent that blurted out some of these responses? These are good directions for future studies. Interestingly, a significant proportion of clinicians believe in the therapeutic benefits of art-based interventions and are open to adopting the same in patient care [37], suggesting that providing answers to these key questions can make art more attractive as a clinician's tool kit.

Clearly, all forms of art seem beneficial. All eight studies included in this review recorded some positive results after art interventions. All age groups can benefit from art. By the nature of the receptor for sound waves and vibration, it is not out of order to start investigating the mechanism of action of art from the nervous system, but the brain is a very complex organ. While the exact mechanism of action has not been elucidated, we can make a few guesses based on the range of responses and our traditional understanding of the activities of neurotransmitters and receptors [38,39]. This is not only for its pedagogic value but for the hope it brings to the future of therapeutic. 

Overall, there seems to be a general consensus on the benefits of art in medicine. One can say without ambiguity that arts-based interventions do have some benefits in clinical practice.

## Conclusions

The ultimate goal of this review was to attempt a meta-analysis of state of the art based intervention in Nigeria's healthcare delivery system. However, given the constraints on the available data, we have exercised caution to avoid making incorrect assertions. Having said that, within the frame of reasonable scientific deduction, there is evidence that art interventions have a role in healthcare delivery in Nigeria. However, much still needs to be done before art-based intervention becomes fully integrated into modern clinical practice. Some of these are general issues, while a few others are local. Locally, the study population is small. Much research on this topic needs to be done on the quality of research. The volume and diversity of the work in this area also need improvement. On a much larger scale, just like any device, material, or substance to be introduced as therapy or its alternative, more quantitative evidence is required on effect, response, pattern, and potential harm in a way that allows for meta-analysis and ease of integration. Increased funding for local art in medicine programs and research is essential to drive these changes, much as policy programs focused on clear-cut implementation plans for Nigeria's healthcare facilities.
